# Investigation of the gene co-expression network and hub genes associated with acute mountain sickness

**DOI:** 10.1186/s41065-020-00127-z

**Published:** 2020-04-16

**Authors:** Yue Chang, Jiange He, Jiqiang Tang, Kai Chen, Zhenguo Wang, Qun Xia, Hai Li

**Affiliations:** 1 Department of Hepatopancreatobiliary and Splenic Medicine, Characteristic Medical Center of People’s Armed Police Force, Tianjin, 300162 China; 2 Tianjin Key Laboratory of Hepatopancreatic Fibrosis and Molecular Diagnosis and Treatment, Tianjin, 300162 China; 3 Institute of Special War Trauma Emergency Technology, Characteristic Medical Center of People’s Armed Police Force, 220 Chenglin Road, Hedong District, Tianjin, 300162 China; 4 Department of Orthopaedics, Characteristic Medical Center of People’s Armed Police Force, Tianjin, 300162 China; 5 Division of Gastroenterology and Hepatology, Tianjin Xiqing Hospital, No.403 Xiqing Road, Xiqing District, Tianjin, 300380 China

**Keywords:** High altitude, Hypoxia, Gene, Acute mountain sickness, Co-expression network, Pathway

## Abstract

**Background:**

Acute mountain sickness has become a heavily researched topic in recent years. However, the genetic mechanism and effects have not been elucidated. Our goal is to construct a gene co-expression network to identify the key modules and hub genes associated with high altitude hypoxia.

**Results:**

The GSE46480 dataset of rapidly transported healthy adults with acute mountain sickness was selected and analyzed by weighted gene co-expression network analysis (WGCNA) to construct a co-expression network. The Gene Ontology (GO) and Kyoto Encyclopedia of Genes and Genomes (KEGG) enrichment analysis of the data set were carried out using Database for Annotation Visualization and Integrated Discovery (DAVID), and the hub genes were selected. We found that the turquoise module was most significantly correlated with acute mountain sickness. The functional enrichment analysis showed that the turquoise module was related to the apoptotic process, protein transport*,* and translation processes. The metabolic pathway analysis identified hsa03010:ribosome and hsa04144:endocytosis as the most important pathways in the turquoise module. Ten top 10 hub genes (M*RPL3, PSMC6, AIMP1, HAT1, DPY30, ATP5L, COX7B, UQCRB, DPM1, and COMMD6*) for acute mountain sickness were identified.

**Conclusion:**

One module and 10 hub genes were identified, which were related to acute mountain sickness. The reference provided by this module may help to elucidate the mechanism of acute mountain sickness. In addition, the hub genes may be used in the future as a biomarker and therapeutic target for accurate diagnosis and treatment.

## Background

The plateau environment is a special ecological environment with low oxygen and low pressure [1]. Approximately, 40 million people venture into high-altitude regions for leisure or work each year [2]. Even with adequate acclimatization, the low oxygen concentration is an inevitable response that multiple organs of the body are in a state of hypoxia at a new altitude in the first 1–2 days [3]. High altitude illness encompasses a multiple spectrum of hypoxemia from acute mountain sickness to high altitude pulmonary edema (HAPE) or high altitude brain edema (HACE) [4–9]. Acute mountain sickness is characterized by a decrease in oxygen saturation, dizziness, lightheadedness, fatigue, sleep disturbance, gastrointestinal symptoms [4]. Interestingly, people who have lived on the plateau for a long time can adapt to such a low-oxygen environment. These people are thought to be more adapted to such an oxygen-deficient environment after years of genetic selection [10, 11]. The adaptation e has been speculated to be related to multiple genes.

In late years, gene expression profile, widely applied in the study of pathogenic genes of a diversity of diseases, is a new popular and useful instrument [12, 13]. Nevertheless, there is still a lack of in-depth analysis of gene expression data related to adaptation to high altitude hypoxia environment, which limits the exploration of key genes for human adapts to the plateau environment. Weighted gene co-expression network analysis (WGCNA) is an increasingly popular method, which can quickly extract gene co-expression modules related to sample features from complex data for subsequent analysis [14]. Simply put, it clusters genes with expression correlation into a module by calculating the expression correlation between genes. Then the correlation between the selected module and the characteristics of the sample (including clinical features, disease progress, therapeutic effect, etc.) was analyzed. It can be seen that WGCNA has built a bridge between sample characteristics and changes in gene expression.

In this written report, eight co-expression modules are constructed by WGCNA, and the interaction between these modules are analyzed in microarray data of acute mountain sickness. Most importantly, 10 hub genes (*MRPL3*, *PSMC6*, *AIMP1*, *HAT1*, *DPY30*, *ATP5L*, *COX7B*, *UQCRB*, *DPM1,* and *COMMD6*) may be related to different phenotypes of acute mountain sickness, which may become therapeutic gene targets for the study of altitude hypoxia adaptation in the future.

## Results

### Microarray data acquisition and gene expression analysis

The keyword “plateau hypoxia or acute mountain sickness” was used to explore GEO datasets in NCBI. The microarray dataset GSE46480 (*https://www.ncbi.nlm.nih.gov/geo/query/acc.cgi?acc=gse46480*) was chosen for this study as it was most relevant to acute mountain sickness.

Subjects were chosen from healthy volunteers participating in the United States Antarctic Program, and were transported from sea-level to an altitude of 3200 m in less than 4 h. A total of 98 subjects (65 males and 33 females, aged from 26 to 50) with acute mountain sickness were identified. All subjects were of high-altitude non-adapted ancestry. Blood samples were taken 3 days after arrival to altitude. Peripheral blood mononuclear cell gene expressions were analyzed from this gene data set, and the sequencing platform was GPL570 ([HG-U133_Plus_2] Affymetrix Human Genome U133 Plus 2.0Array).

**Fig. 1:**
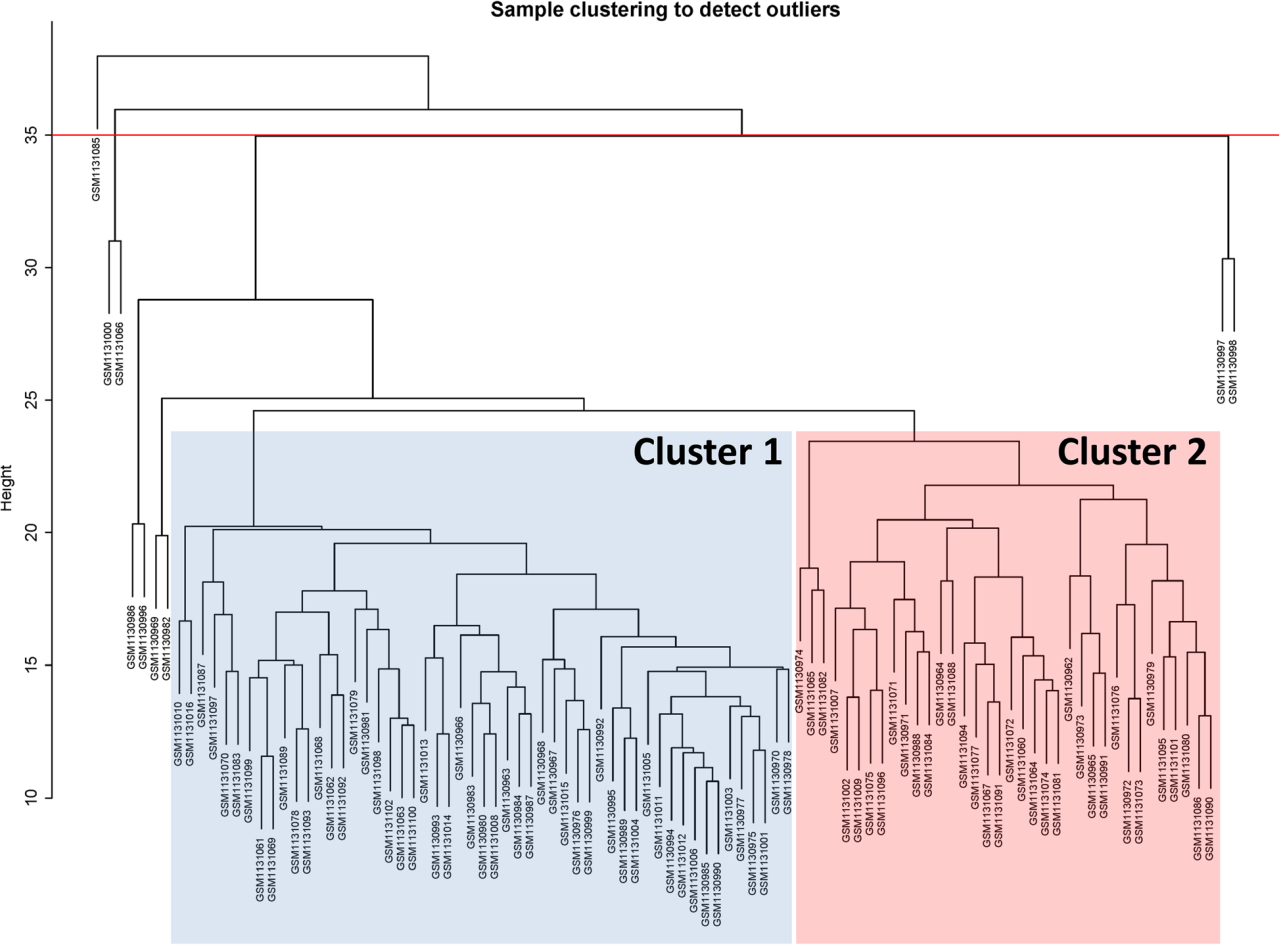
Cluster analysis of high altitude hypoxia samples. Those highest point 4121 genes with the most astounding of high altitude hypoxia samples were analyzed and identify by Weighted Gene Coexpression Network Analysis and *flashclust*. All the samples were grouped into two clusters, cluster 1 (32 samples) and cluster 2 (59samples), respectively. The red line has been used to recognize those outlier samples (GSM1131085, GSM1131000, GSM1131066, GSM1130997, and GSM1130998), as the threshold set at 35

A total of 21,656 gene expression values were obtained from the original data. The top 4121 genes with the highest average expression value were selected for cluster analysis by WGCNA algorithm and *hClust* software package (Fig. 1). When the threshold of the clustering height was determined to be 35, there were 5 outlier samples (GSM1131085, GSM1131000, GSM1131066, GSM1130997, and GSM1130998) in all samples. For the reliability of the module, these outlier samples were eliminated. As shown in Fig. 1, the remaining samples analyzed were divided into two groups (36samples in Cluster 1 and 53 samples in Cluster 2) by Hierarchical cluster clustering. In addition, four samples from the outermost branch (GSM1130986, GSM1130996, GSM1130969, and GSM1130982) were very nearly to two groups also included in this analysis. Finally, only the remaining 89 samples were further analyzed.

### Construction of plateau hypoxia gene co-expression module

**Fig. 2:**
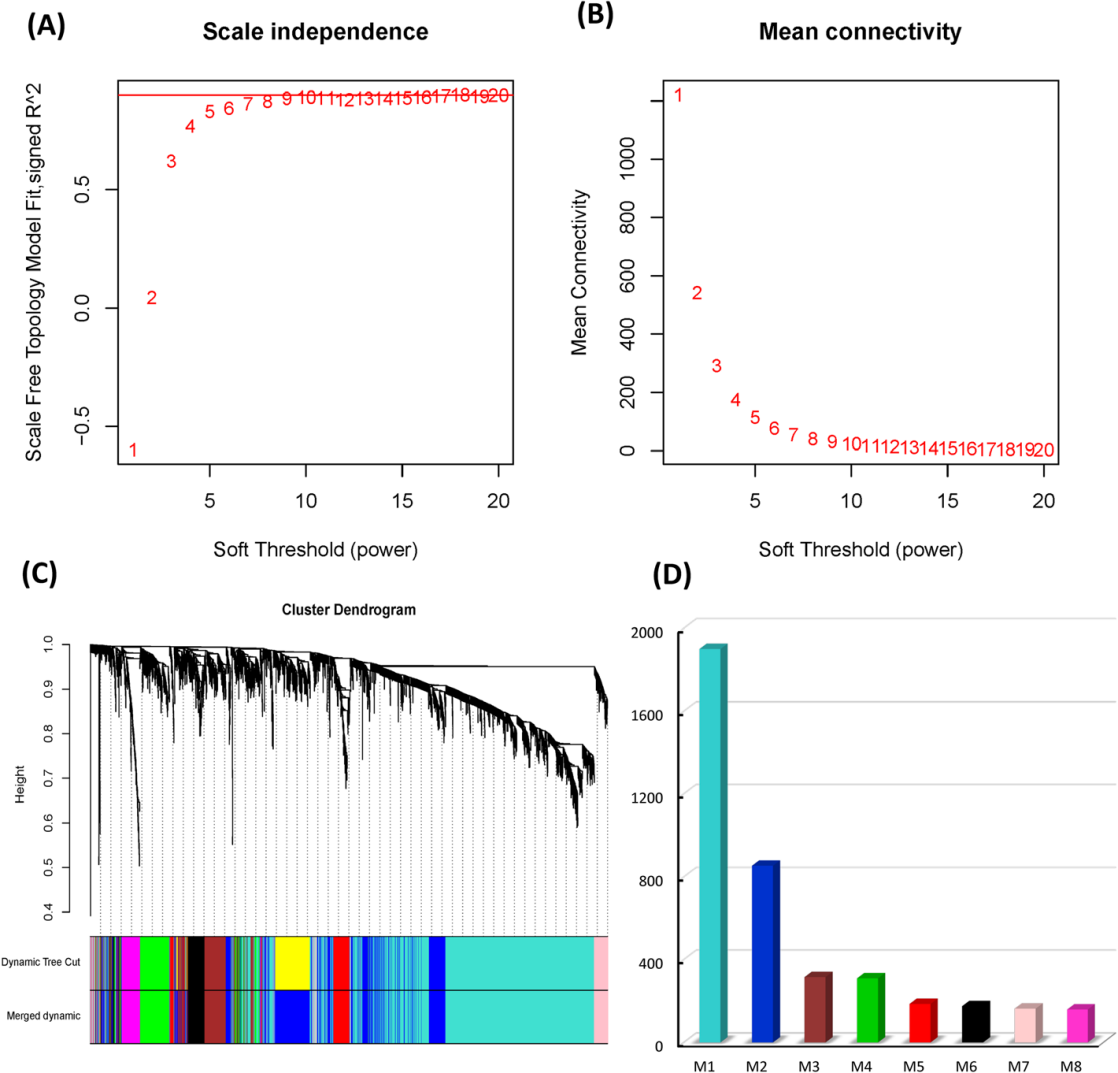
Development of coexpression modules of high altitude hypoxia genes. **a** The effect of different control values of coexpression modules of high altitude hypoxia genes on the scale independence degree. **b** The impact for diverse control worth on the normal connectivity degree for coexpression modules of high altitude hypoxia genes. **c** The constructed co-expression modules from high altitude hypoxia genes by Weighted Gene Coexpression Network Analysis. **d** The amount for genes in distinctive co-expression modules. Each branch represents a different coexpression module. Those symbol M below stands to the module, and the number in the brackets speaks to those amount of genes in the module

The WGCNA algorithm was used to construct the co-expression module from the expression values of 4121 genes. First, the power value was screened out that when the value was 7, the scale independence was 0.85 (Fig. 2a). At the same time, it also had a higher average connectivity value (Fig. 2b). Then, we used 4121 genes with a high apparent value to construct a co-expression module (Fig. 2c). Different modules were sorted from high to low according to the number, and distinguished by different colors. As a result, a total of 8 co-expression modules were constructed. Nevertheless, 55 Genes (1.33%) that cannot be included in any module were placed in the gray module and removed in subsequent analysis. At the same time, the number of genes in each module was analyzed. The average number of genes in the 8 modules was 508 and the median was 248. There were 1901 genes in module 1 (turquoise), 854 genes in module 2 (blue), 315 genes in module 3 (brown), 309 genes in module 4 (green), 187 genes in module 5 (red), 176 genes in module 6 (black), 164 genes in module 7 (pink), and 160 genes in module 8 (magenta).

### Analysis of interaction between co-expression modules

**Fig. 3:**
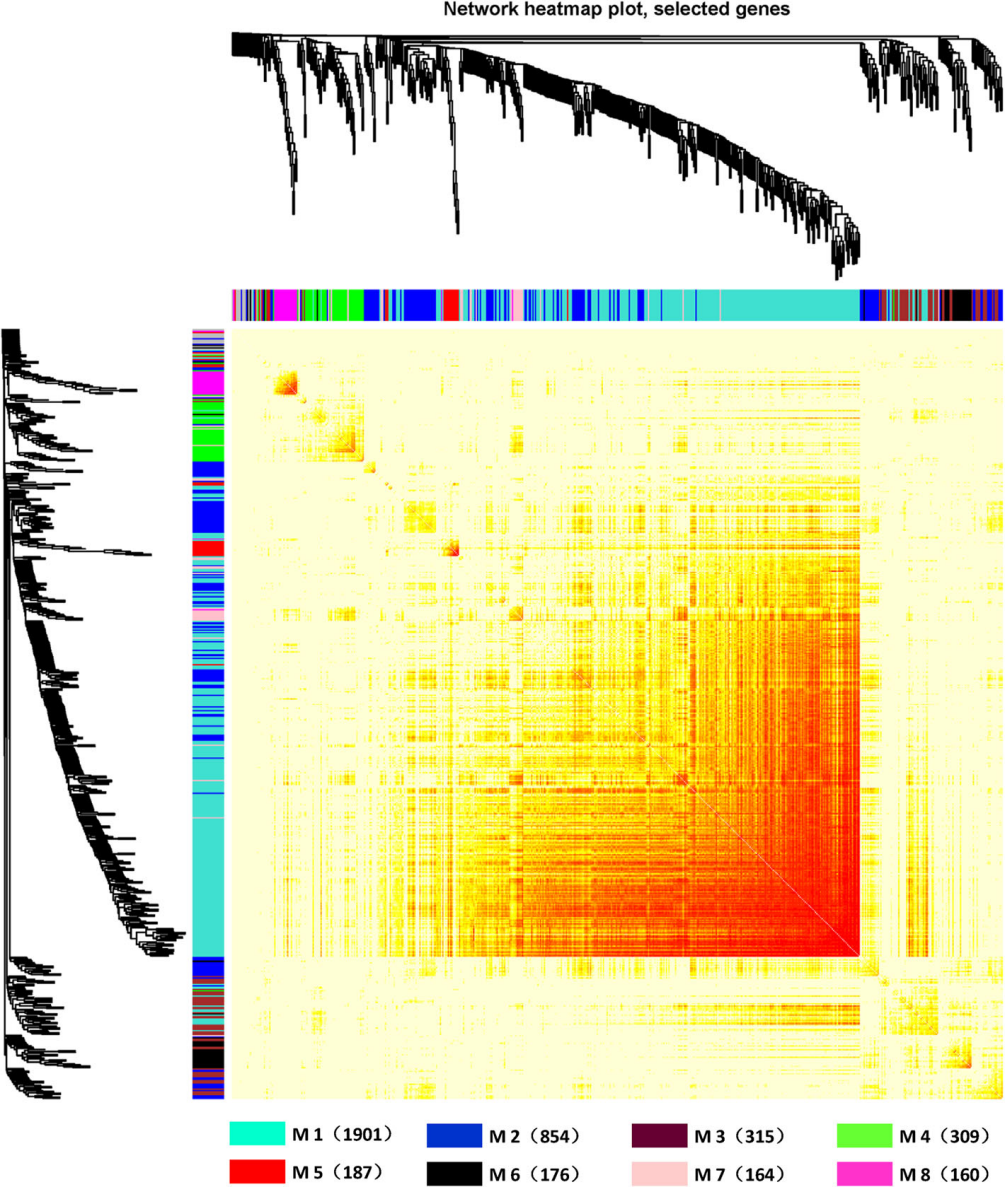
Association analyses for coexpression modules. Distinctive colors on the vertical or horizontal axis stood for distinctive modules. The brilliance for the yellow color in the center stood for the relevance and importance quality among these modules. There have been not any significant critical contrasts of the association among these modules, demonstrating the higher scale independence. Those symbols M stands to the module, and the number in the brackets speaks to those amount of genes in the module

**Fig. 4:**
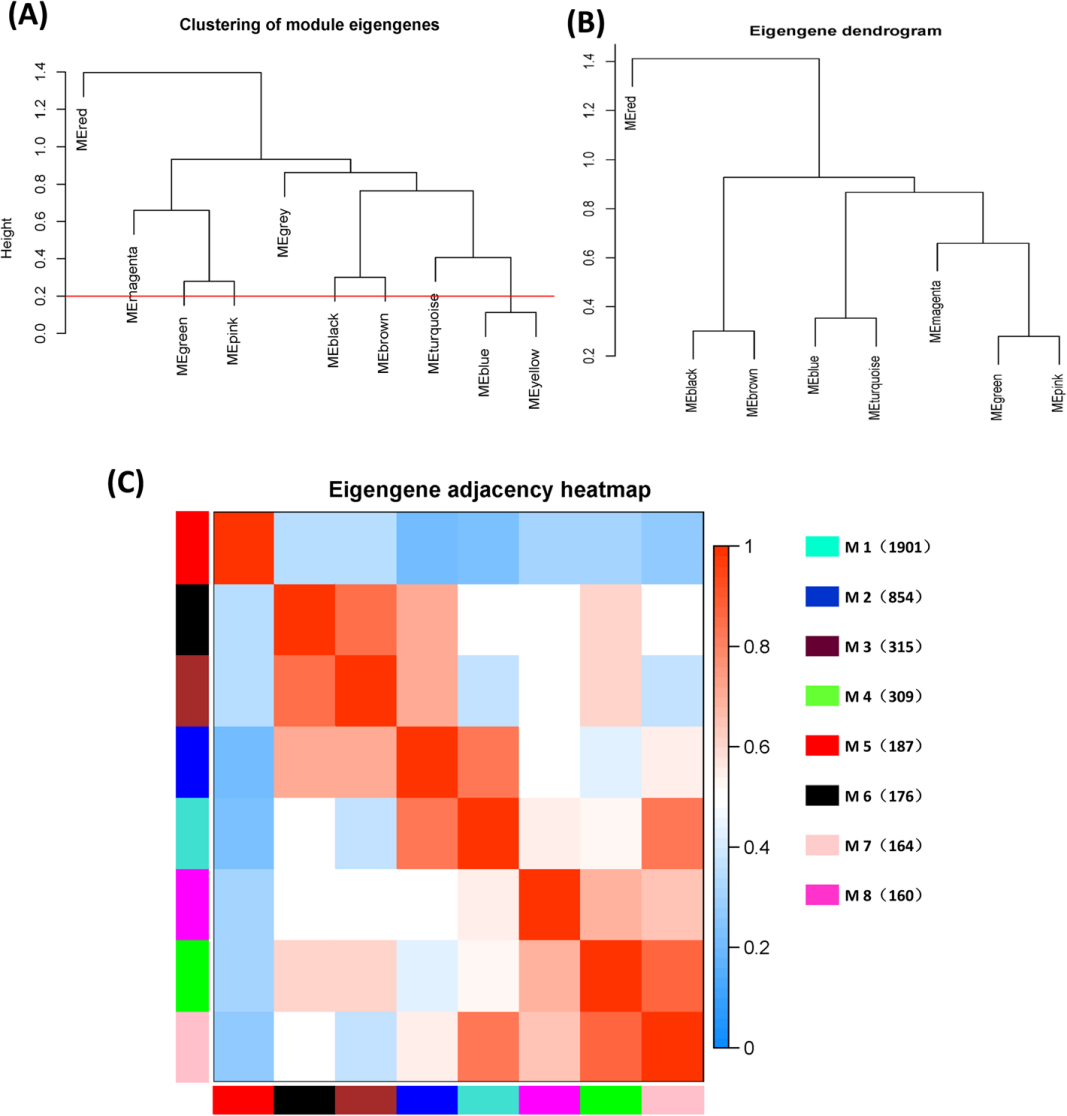
Connectivity examination about eigengenes of distinctive modules. **a** Cluster dissection for eigengenes in the module. **b** Eight modules were produced; MEDissThres was set as 0.2 to merge homologous modules. A hierarchic grouping from module eigengenes that sum up the modules yielded in the grouping examination. **c** Nearness heat map about eigengenes in the module. Two clusters were obtained, including one module (module 5) and seven modules (module 1, 2, 3, 4, 6, 7 and 8). The gradient change of shade from red (1) to blue (0) represents that nearness about eigengenes in distinctive modules from strong to weak. Those symbols M on the straight stands to the module, and the number in the brackets speaks to those amount of genes in the module

A network heat map was drawn to show the relationship between 8 modules (Fig. 3). The results showed that each module and the relative of gene expression in each module were all independent. Then, the eigengenes of each module feature were identified. According to the correlation of eigengenes, clustering was carried out to explore the co-expression similarity of all modules (Fig. 4a and b). The result showed that the eight modules were mainly divided into two clusters. The heat map of the module relationship was drawn showed the same results as above (Fig. 4c).

### Functional enrichment analyzed by GO and KEGG

The functional enrichment analyzed by GO and KEGG was carried out on the 8 modules. There were significant differences in the results of functional enrichment analysis among different modules. Moreover, the top 5 enriched GO terms were presented in Table 1. The genes in module 1 (turquoise) were mainly enriched in the process related to apoptotic process and protein transport, and the key terms were GO:0006915 (apoptotic process), GO: 0015031 (protein transport), and GO:0006412 (translation). Most of the genes in module 2 (blue) were enriched in apoptosis and protein translation, and the most enriched terms were GO:0006915 (apoptotic process) and GO:0006412 (translation). Most of the genes in module 3 (brown) were enriched in the process related to innate immune response and cell adhesion, and the key terms were GO:0045087 (innate immune response), GO:0098609 (cell-cell adhesion) and GO:0006886 (intracellular protein transport). The genes in module 4 (green) were mainly enriched in signal transduction and inflammatory response, and the most abundant GO terms were GO:0007165 (signal transduction) and GO:0006954 (inflammatory response). The genes in module 6 (black) were mostly enriched in the process of mRNA splicing and intracellular signal transduction, and the most important GO terms were GO:0000398 (mRNA splicing, via spliceosome) and GO:0035556 (intracellular signal transduction). The genes in module 7 (pink) were mainly enriched in GO:0006915 (apoptotic process) and GO:0043066 (negative regulation of apoptotic process). The genes in module 8 (magenta) were mainly enriched in GO:0051607 (defense response to virus), GO:0060337 (type I interferon signaling pathway), and GO:0045087 (innate immune response). Besides, there was no obvious enrichment of terms in module 5 (red).

**Table 1:** GO enrichment for the genes in the coexpression modules of glioma

Module	Term	Count	%	PValue
M1	GO:0006915~apoptotic process	87	4.71	2.40E-05
	GO:0015031~protein transport	66	3.57	1.90E-05
	GO:0006412~translation	63	3.41	5.96E-12
	GO:0098609~cell-cell adhesion	62	3.35	3.69E-10
	GO:0051301~cell division	56	3.03	2.74E-04
M2	GO:0006915~apoptotic process	42	5.12	0.001513
	GO:0006412~translation	41	5.00	2.32E-12
	GO:0006413~translational initiation	36	4.39	1.28E-17
	GO:0000398~mRNA splicing, via spliceosome	35	4.27	2.54E-10
	GO:0016032~viral process	35	4.27	4.94E-07
M3	GO:0045087~innate immune response	19	6.11	6.26E-04
	GO:0098609~cell-cell adhesion	15	4.82	3.26E-04
	GO:0006886~intracellular protein transport	13	4.18	9.88E-04
	GO:0050900~leukocyte migration	12	3.86	1.02E-05
	GO:0008360~regulation of cell shape	12	3.86	3.72E-05
M4	GO:0007165~signal transduction	46	15.23	5.41E-08
	GO:0006954~inflammatory response	33	10.93	2.59E-14
	GO:0045087~innate immune response	24	7.95	6.95E-07
	GO:0006955~immune response	20	6.62	6.90E-05
	GO:0006915~apoptotic process	20	6.62	0.002587
M6	GO:0000398~mRNA splicing, via spliceosome	9	5.49	5.01E-04
	GO:0035556~intracellular signal transduction	9	5.49	0.0183
	GO:0006357~regulation of transcription from RNA polymerase II promoter	9	5.49	0.029314
	GO:0006397~mRNA processing	8	4.88	6.98E-04
	GO:0015031~protein transport	8	4.88	0.044714
M7	GO:0006915~apoptotic process	12	7.64	0.00608
	GO:0043066~negative regulation of apoptotic process	10	6.37	0.011294
	GO:0007264~small GTPase mediated signal transduction	9	5.73	8.48E-04
	GO:0042981~regulation of apoptotic process	6	3.82	0.028884
	GO:0002223~stimulatory C-type lectin receptor signaling pathway	5	3.18	0.010221
M8	GO:0051607~defense response to virus	31	19.87	6.03E-32
	GO:0060337~type I interferon signaling pathway	24	15.38	3.00E-32
	GO:0045087~innate immune response	21	13.46	5.29E-10
	GO:0009615~response to virus	19	12.18	1.42E-18
	GO:0045944~positive regulation of transcription from RNA polymerase II promoter	18	11.54	0.003564

The signaling pathways analyzed by KEGG were summarized in Table 2. The result showed that a total of 1901 genes were recognized as the genes with high absolute correlations in module 1 and mainly enriched in hsa03010: Ribosome and hsa04144: Endocytosis. Module 2 was mainly enriched in the pathways hsa05016: Huntington’s disease and hsa03010: Ribosome. Module 3 was mainly enriched in metabolic pathways hsa04145: Phagosome, hsa05168: Herpes simplex infection, and hsa04144: Endocytosis. Module 4 was mainly enriched in hsa04380: Osteoclast differentiation. Module 6 was mainly enriched in hsa04010: MAPK signaling pathway and hsa03040: Spliceosome. Module 7 was enriched in pathways hsa05200: Pathways in cancer. Module 8 was enriched in hsa05162: Measles and hsa05164: Influenza A. There was no significantly enriched term in module 5.

**Table 2:** KEGG pathway enrichment for the genes in the coexpression modules

Module	Term	Count	%	PValue
M1	hsa03010:Ribosome	55	2.98	5.82E-18
	hsa04144:Endocytosis	53	2.87	2.69E-06
	hsa05016:Huntington’s disease	48	2.60	1.8E-07
	hsa05012:Parkinson’s disease	47	2.54	1.18E-11
	hsa00190:Oxidative phosphorylation	44	2.38	6.04E-11
M2	hsa05016:Huntington’s disease	31	3.78	6.94E-08
	hsa03010:Ribosome	29	3.54	3.32E-10
	hsa05012:Parkinson’s disease	27	3.29	1.97E-08
	hsa05010:Alzheimer’s disease	26	3.17	2.25E-06
	hsa00190:Oxidative phosphorylation	24	2.93	3.91E-07
M3	hsa04145:Phagosome	17	5.47	2.48E-07
	hsa05168:Herpes simplex infection	16	5.14	1.63E-05
	hsa04144:Endocytosis	14	4.50	0.003325
	hsa04142:Lysosome	13	4.18	1.79E-05
	hsa04141:Protein processing in endoplasmic reticulum	13	4.18	0.000453
M4	hsa04380:Osteoclast differentiation	19	6.29	3.23E-10
	hsa05152:Tuberculosis	16	5.30	6.22E-06
	hsa04145:Phagosome	15	4.97	4.18E-06
	hsa05140:Leishmaniasis	13	4.30	3.15E-08
	hsa05164:Influenza A	13	4.30	0.00039
M6	hsa04010:MAPK signaling pathway	7	4.27	0.027535
	hsa03040:Spliceosome	6	3.66	0.007182
	hsa05164:Influenza A	6	3.66	0.021085
	hsa04015:Rap1 signaling pathway	6	3.66	0.04254
	hsa00310:Lysine degradation	5	3.05	0.001245
M7	hsa05200:Pathways in cancer	11	7.01	0.00439
	hsa05152:Tuberculosis	9	5.73	0.000293
	hsa04071:Sphingolipid signaling pathway	7	4.46	0.001042
	hsa05161:Hepatitis B	7	4.46	0.002742
	hsa04650:Natural killer cell mediated cytotoxicity	6	3.82	0.006497
M8	hsa05162:Measles	13	8.33	5.71E-09
	hsa05164:Influenza A	12	7.69	1.01E-06
	hsa05168:Herpes simplex infection	11	7.05	1.21E-05
	hsa05160:Hepatitis C	10	6.41	6.11E-06
	hsa04978:Mineral absorption	6	3.85	7.03E-05

### Identification of hub genes

**Fig. 5:**
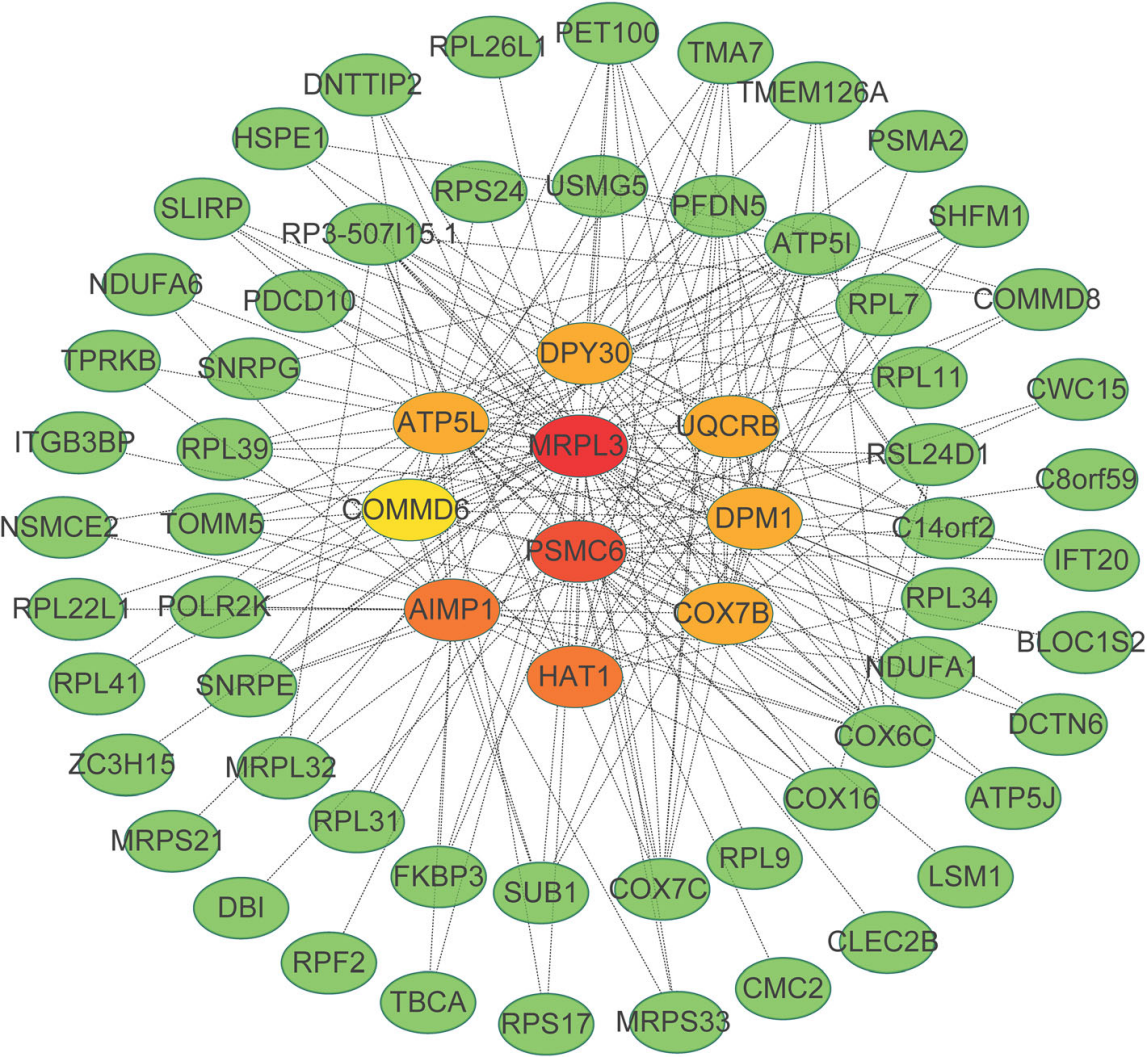
GO enrichment analysis of high altitude hypoxia genes in co-expression modules. The top 10 hub genes constructed in network in the turquoise module. Nodes stood for genes and lines represented the interactions of genes

We visualized the turquoise module as networks in Cytoscape and screened out the top 10 genes ranked by Degree method. Figure 5 shows the top 10 hub genes in the turquoise module. They were *MRPL3*, *PSMC6*, *AIMP1*, *HAT1*, *DPY30*, *ATP5L*, *COX7B*, *UQCRB*, *DPM1*, and *COMMD6* in the turquoise module.

## Discussion

In this study, we found 8 gene modules related to acute mountain sickness by WGCNA method. Several core pathways and 10 hub genes were found by GO and KEGG. To the best of our knowledge, this is the first study focus on the hub genes related to acute mountain sickness. Our results may provide new help for the prevention and treatment of acute mountain sickness in future research.

Generally, the physical symptoms such as fatigue or headache may develop ascending rapidly to altitudes > 2500 m [15, 16]. Moreover, the physical symptoms and impaired alveolar oxygen diffusion tend to be more severe over 4500 m [17]. A real-world study from Harrison MF et al. reported that subjects with acute mountain sickness had lower oxygen saturation [9]. Indeed, people exposed to high altitudes may have acute hypoxia. Interestingly, not all new entrants to the high altitude develop AMS, which has an incidence of 30 to 69% based on the altitude [18–20]. Additionally, it deemed to individual and gene differences are genetically related [21].

Instead of focusing only on differentially expressed genes, WGCNA can recognize the set of genes of interest using the information of specific or all genes and makes a significant association analysis with phenotypes [22]. By using 98 samples, 8 co-expression modules with relative independence were constructed. The interaction between modules is co-expressed by the thermographic reaction. Module 1 (turquoise) is considered to be the core module, and we further analyzed it.

Functional enrichment by GO suggested that GO:0006915 (apoptotic process), GO: 0015031 (protein transport) and GO:0006412 (translation) in module 1 (turquoise) were valuable data and important components in cellular metabolism in high altitude circumstances. Noteworthy, mitochondrial dysfunction and endothelial barrier dysfunction play a key role in hypoxic diseases, especially cute mountain sickness, HAPE and other plateau hypoxia-related diseases. By differential proteomic and immunoassay techniques, 30% drops of glycolytic enzymes and of muscular creatine kinase abundance were found in subjects up to 8848 m. The impaired tricarboxylic acid (TCA) cycle and mitochondrial dysfunction were also found [23]. By reviewing the literature, we found that the apoptotic process [24], protein transport and translation are impaired in hypoxic conditions [25, 26]. Recently, Tsai SH et al. found that hypoxia leads to hypoxia-inducible factor-1α (HIF-1α) expression and endothelial barrier dysfunction in healthy adult volunteers ascend to 3100 m height [27]. Yang YD et al. suggested that cell apoptosis is related to mitochondria-associated membranes in hypoxia-induced endothelial injury [28]. This evidence verifies the clinical value of functional enrichment by GO in Module 1.

The signaling pathways identified as hsa03010:Ribosome and hsa04144:Endocytosis. This conclusion is supported by a recent plasma proteomic study in the plateau of volunteers [29]. After 9 h of hypoxia, proteins related to the TCA cycle and ribosomes were significantly reduced in acute mountain sickness resistant individuals [30]. Furthermore, autophagy increase is related to endocytosis after the intestinal failure in Wistar rats acutely exposed to hypobaric hypoxia in plateau stress. Therefore, it is not queer to conclude that the mTOR signaling pathway participates in hypoxic memory injury in plateau and may be a potentially important therapeutic target for low-pressure and low-oxygen [31].

Moreover, 10 genes were screened out as hub genes. Many of them have been well documented to be associated with hypoxia such as *AIMP1* [32], *HAT 1* [33], and *DPY30* [34] as well. Hub genes are highly connected genes by the WGCNA and the by Cytoscape software considered as functionally significant. So the hub genes can be different from differential genes. These novel hubs gene may use as a biomarker or therapeutic target for accurate diagnosis and treatment in acute mountain sickness in the future.

## Conclusion

All in all, our fruitful work gives an explanation of the gene co-expression network and hub genes in acute mountain sickness by functional enrichment and metabolic pathway analysis. The turquoise module was most significantly correlated with acute mountain sickness. And the hsa03010: Ribosome and hsa04144: Endocytosis were screened as the most important pathways while 10 hub genes were identified. The hub genes may be used in the future as a biomarker and therapeutic target for accurate diagnosis and treatment. Of course, more research with acute mountain sickness is still needed in the future, especially in terms of the function of different genes.

## Materials and methods

### Gene expression analysis of acute mountain sickness

The microarray data were downloaded from the genome expression summary dataset on the *https://www.ncbi.nlm.nih.gov/geo/* website with the keyword “altitude hypoxia *or* acute mountain sickness”, meanwhile “*Homo sapiens*”, “Expression profiling by array” were selected. The signal information of the probe provided in the dataset was downloaded. Then, matched the probe with the corresponding gene used the annotation information provided by the record. The number of genes was counted under different gene expression thresholds. WGCNA was used to evaluate the gene expression value and selected the appropriate threshold. In addition, the *hclust* toolkit in R language was used to cluster the samples under the appropriate threshold [35].

### Construction of gene co-expression module at in acute mountain sickness

The weighting value in the construction of the module was screened out by the WGCNA algorithm. Gradient tests were used to analyze the scale independence and average connectivity of the module at different power values (from 1 to 20). The soft-thresholding power was selected when the degree of scale independence was set to 0.85.

Then at the soft-thresholding power value selected above, the co-expression module was built using the WGCNA algorithm. In addition, the corresponding gene information in each module was extracted. In order to obtain more accurate results, the minimum number of genes in the module was set to 40.

### Interaction analysis of hypoxia gene co-expression module in acute mountain sickness

The relationship between the co-expression of acute mountain sickness was analyzed by the WGCNA algorithm. WGCNA was carried out by R language (http://R-project.org/), and the intensity of these interactions was reflected by heat maps.

### Functional enrichment analysis of genes in hypoxia co-expression module of acute mountain sickness

The co-expression modules were sorted according to the number of genes in them. After that, the functional enrichment analysis of these genes was carried out. The genetic information in the corresponding module was used in Kyoto Encyclopedia of Genes and Genomes (KEGG) and gene ontology (GO) path analysis. The related annotation, visualization, and integration discovery were carried out if the corrected *p* < 0.05. If the number of records exceeds five, the top five records were selected for further analysis.

### Identification of hub genes

Hub genes were visualized in networks by *Cytoscape* (*version* 3.7.0). Additionally, the top 10 hub genes in networks were identified by the degree and considered as functionally significant.

## Data Availability

The profiles of GSE46480 can download in GEO datasets (https://www.ncbi.nlm.nih.gov/geo/query/acc.cgi?acc=gse46480). The algorithm can be obtained by the corresponding author Hai Li via Email (haili_tj@sina.com).

## Abbreviations

DAVID: Database for Annotation Visualization and Integrated Discovery
GO: Gene Ontology
HACE: High altitude brain edema
HAPE: High altitude pulmonary edema
KEGG: Kyoto Encyclopedia of Genes and Genomes
TCA: Tricarboxylic acid
WGCNA: Weighted gene co-expression network analysis
